# Mental health of children and young people in the WHO Europe region

**DOI:** 10.1016/j.lanepe.2025.101459

**Published:** 2025-10-06

**Authors:** Anna Tarasenko, Gisel Josy, Hellen Minnis, Jennifer Hall, Andrea Danese, Jennifer Y.F. Lau, Samuele Cortese, Argyris Stringaris, Cassie Redlich, Dennis Ougrin

**Affiliations:** a United Nations Office on Drugs and Crime, Kyiv, Ukraine; b Service User, UK; c University of Glasgow, Glasgow, UK; d World Health Organisation, Athens, Greece; e King's College London, London, UK; f Queen Mary University of London, London, UK; g University of Southampton, Southampton, UK; h University College London, London, UK; i World Health Organisation, Copenhagen, Denmark

**Keywords:** Global mental health, Children and young people, Review

## Abstract

Most mental health disorders start before adulthood. They are highly prevalent, disabling and often treatable. This Series paper discusses the current problems that contribute to the growing child, adolescent and youth mental health crisis in Europe. These include the impact of the COVID-19 pandemic, escalating military conflicts, the climate crisis, and unregulated digital environments. Mental health problems in youths are further compounded by a changing landscape of how children and young people engage with the world, healthcare and other services. We outline a comprehensive list of recommendations to address mental health problems through the integration of creative prevention and treatment approaches with the help of community-based services and support systems, as well as robust research and implementation strategies to ensure cost-effective, evidence-based care.

## Introduction

Globally, there have been calls to urgently address the growing child, adolescent and youth^1^ mental health crisis.^2^ At the first meeting of the WHO Pan-European Mental Health Coalition, improving the quality of child and youth mental health care across the Region was listed as a key priority.^3^ Childhood and adolescence provide a crucial window of opportunity to improve mental health across the lifespan, with cumulative benefits and avoidance of the greater challenge and expense of intervening later in life.^4^ Most mental health disorders begin in childhood and adolescence, with the peak age of onset at 14.5 years.^5^ Earlier onset of psychopathology is also associated with greater chronicity and complexity of psychopathology later in life.^6^ Globally, about 300 million of 2.5 billion people aged 5–24 have a mental health disorder, and these account for about 30 million of 150 million years lived with disability.^7^

There is evidence of rising rates of anxiety^8^ and depression during childhood and adolescence, and concern about an increase in the diagnoses of neurodevelopmental conditions such as ADHD^9^ and autism,^10^ with significant variations across the region. Available data show declining self-reported well-being in childhood and adolescence.^11^ Despite a remarkable overall reduction in suicide across the region over the last decade, especially in southern Europe, some countries like the UK show a consistent annual increase in suicide rates of up to 2.5% in boys and 8.5% in girls from 1990 to 2020.^12^ Studies have shown that feelings of hopelessness, isolation, and the pressures of academic and social performance contribute to these tragic outcomes.^13^ It is estimated that 59% of 16–25-year-olds are very or extremely worried about the state of the planet.^14^ Yet, more studies need to be conducted in primary school-aged children when these worries arise, and could lead to identifying remedial action.^15^

**Key messages**

• There is a growing child, adolescent, and youth mental health crisis in the WHO Europe region. Yet, childhood and adolescence provide a crucial window of opportunity to improve mental health across the whole lifespan.
• The main contributors to the increasing mental health needs are the impact of the COVID-19 pandemic, escalating military conflicts, the climate crisis and unregulated digital environments. These are further compounded by a changing landscape of how children and young people engage with healthcare and other services.
• Addressing mental health problems in youths requires a holistic approach, from treatment to prevention, from analogue to digital and from inpatient to community.

Panel 1 **Recommendations to improve the mental health of children and young people**

• A holistic approach that includes preventive measures in schools, equitable access to mental health services, ending wars, and efforts to tackle the root causes of socioeconomic inequality.
• Expanding community care services and community-based support systems is vital to address gaps in service provision.
• Embracing the use of technology through the co-development and co-design of regulations and interventions, together with service users, is required.
• A comprehensive, overarching framework for quality improvement of delivered care should be implemented across the WHO Europe region.
• Repurposing existing drugs and tailoring trials to paediatric conditions are key strategies to improve treatment options for children and adolescents.
• Robust research and implementation strategies can help to solve the implementation crisis.

**Search strategy and selection criteria**

We based our search strategy on three reviews spanning PubMed, Web of Science, PsychINFO, Embase searched from inception to 05/2020–01/2022-with the search terms as follows:

A. “age at onset” (topic) and “mental disorder” (topic), plus “birth cohort” (topic) and “mental disorder” (topic), plus “incidence” (topic) and “mental disorder” (topic)^5^;
B. ((maltreatment) OR (child∗ abuse) OR (child∗ neglect) OR (child∗ trauma) OR (child∗ advers∗) OR (harsh punishment) OR (institutional deprivation) OR (early life deprivation) OR (early life stress) OR (victim∗) OR (institutionali#ation))
AND ((mental health) OR (mental illness) OR (psychopathol∗) OR (psychiatric) OR (internali∗) OR (externali∗) OR (depress∗) OR (anxi∗) OR (panic) OR (obsessive compulsive) OR (self inj∗) OR (self harm∗) OR (suicid∗) OR (eating disorder∗) OR (schiz∗) OR (psychotic) OR (psychosis∗) OR (bipolar) OR (ADHD) OR (attention deficit hyperactivity disorder) OR (attention) OR (hyperactiv∗) OR (neurodev∗) OR (conduct) OR (antisocial) OR (anti social) OR (crim∗) OR (substance) OR (alcohol) OR (drug) OR (cannabis)).
AND ((twin∗) OR (sibling∗) OR (children of twins) OR (natural experiment) OR (adopt∗) OR (propensity score) OR (inverse probability weight∗) OR (matching) OR (fixed effects) OR (quasiexperiment∗ OR quasi experiment∗) OR (causal∗) OR (genetically sens∗) OR (genetically inform∗) OR (instrumental variable∗) OR (interrupted time series analysis) OR (Mendelian randomi#ation) OR (regression discontinuity) OR (experience sampl∗) OR (ecological momentary assessment∗) OR (difference in difference∗)).^41^

C. baby OR babies OR infant OR infants OR infancy OR child OR children OR puberty OR adolescent OR adolescents OR adolescence OR youth OR toddler OR childhood OR newborn OR newborns, orphanage OR orphanages OR orphan OR orphans OR “residential care” OR “institutional care” OR “congregate care”, to which we added the following exclusion terms: “orphan drug”, “orphan nuclear”, orphan parvovirus”, “orphan disease”, “orphan pattern”, “orphan strain”, “orphan gene”, “diagnostic orphan”, “therapeutic orphan”, “orphan virus”, boarding, “old people home”, “qualitative study”; development OR developmental OR growth OR height OR “failure to thrive” OR nutrition OR health OR cognitive OR cognition OR brain OR neurolog∗ OR neural OR disorder OR disorders OR “quality of life” OR neurodevelopment∗ OR socioemotional OR “behavior problem” OR “problem behaviors” OR externalizing OR internalizing OR mortality OR morbidity OR delinquen∗ OR criminali∗ OR “psychosocial development” OR psychopatholog∗ OR depress∗ OR adhd OR inattention OR autism OR “atypical development” OR education OR IQ OR motivation OR attachment OR cortisol OR dwarfism OR infect∗ OR medical OR disease OR psychophysiology OR immune OR wellbeing OR resilien∗ OR respiratory OR endocrine OR hormonal OR “stress regulation” OR “executive function” OR “reproduction fitness” OR offspring.^80^

Panel 2 **Service user's perspective**

“Having struggled greatly with my mental health during my adolescence and early adulthood, I spent many years in psychiatric units across the UK. Hence, I cannot emphasise enough how life-changing early intervention can be for a young person. In my case, the intervention came just before it was too late. Had it happened sooner, perhaps admission to inpatient units may have been avoided.

At the age of 15, I was diagnosed with Anorexia Nervosa while on an acute paediatric ward. My deteriorating physical health and diminished quality of life had reached a breaking point. However, it should have never got to that stage. Despite my multiple attendances to the GP prior to my admission, my situation was constantly dismissed as a ‘phase all girls go through.’ This was an oversight that cost the next six years of my life. I exhausted various treatments, including medications, therapies, and admissions to both inpatient and day-patient specialist services. During this time, I experienced both CAMHS and adult mental health services. Despite the intensity and promise each treatment offered, it all felt futile.

My final admission coincided with the COVID-19 pandemic. Being 4 h away from family and friends was hard enough, but the pandemic made it almost impossible to see them. The isolation and relentless constrictions, both from the pandemic and being a sectioned patient, felt like imprisonment.

During my time in inpatient wards, I learned numerous ways to cope, some of which were healthy, but many of which were not. For me, self-harm and suicidal ideations became my haven when life felt unbearable. This personal experience links well with the broader discussion around elevated rates of self-harm and suicide in the younger generation, mentioned earlier. Beyond the statistics, in my opinion, self-harm acts as a coping strategy for distress that you struggle to vocalise or which goes unheard. Within inpatient units, self-harm can be normalised due to it being perceived as a marker of illness and the medical response it elicits. It is not an attention-seeking behaviour but rather a behaviour that requires attention and help with the deeper, underlying issues.

Based on all the treatment I've received over the years, I believe that what led me to remission was the incredible community-based care I had alongside my drive to get into medical school. As an inpatient, you often lose sight of the outside world and the sense of a life beyond your illness. It was only when I realised that it wasn't all a farfetched reality and I learned how to apply the skills I was taught in therapy to real-world circumstances that I actively chose recovery. I am a strong believer that true recovery rarely happens in mental health wards. These wards are there to treat you acutely, but the commitment to recovery for diagnoses such as mine has to come from within.

Hence, this supports the idea that moving away from institutional care and prioritising community care is necessary. Ultimately, recovery should take place in the environment where the challenges began so that individuals are equipped with the skills needed to face similar challenges in the future. Community teams provide ongoing support needed to help individuals manage their illness in their home environment, thus reducing the risk of institutionalisation.

To conclude, future interventions for the mental health of young people should focus on more community-based treatment and addressing the underlying factors contributing to their deteriorating mental health. This is far better than simply talking through the issues. This approach would pave the way for a more sustainable and effective recovery.”

In the WHO Europe region, a pan-European collaborative approach to addressing the rapid decline in child and adolescent mental health is essential and requires adjustments for regional aspects. This work is led by the WHO Regional Office for Europe, which coordinates the work of a number of stakeholders in all countries of the region.^16^

## Current challenges

### Impact of the COVID-19 pandemic

The pandemic severely disrupted the lives of children and adolescents, affecting their socialisation, education, and mental health.^8^ Lockdowns created an extraordinary social change during which parents and children were forced to live in close proximity without wider social support. Extended lockdowns, school closures, and social distancing measures led to increased feelings of isolation, loneliness,^17^ anxiety, and uncertainty. COVID-19 measures varied between the different countries, with the number of school closure days ranging from zero in Finland and Sweden to 341 days in Italy.^18^ 17% of children in Republic of Moldova reported feeling negative effects of COVID-19 on their mental health and wellbeing in comparison to 38% and 37% in the Republic of Ireland and the UK.^19^ Studies indicate an increase in referrals for psychiatric disorders following the lockdown, including a substantial rise in self-harm presentations to emergency departments.^20^ At the same time, the post-COVID-19 changes in services created opportunities to develop telepsychiatry^21^ and improve access to digital services.^22^

### Social media and digital technology

While digital technology and social media provide avenues for connection, their overuse has been established as a possible risk factor for poor mental health outcomes in children and adolescents.^23^ However, the evidence remains mixed. Concerns include overexposure to war-related imagery and inappropriate sexual content. The Health Behaviour in School-aged Children survey report from 2021/22 measured the use of digital technologies amongst young people across Europe, Central Asia, and Canada, including social media use (SMU), continuous online social contact, and gaming behaviours. In comparison to the 2017/2018 survey, the 2021/2022 findings showed an increased prevalence of problematic SMU—rising from 7% to 11% of young people, with the highest increase in Romania. The lowest prevalence of SMU was found in the Netherlands, 5% prevalence and the highest in Romania–22%.

Cyberbullying,^24^ body image concerns^25^ and the pressure to conform to online standards and social comparisons contribute to increasing levels of anxiety and depression. The constant exposure to unrealistic portrayals of life can exacerbate feelings of inadequacy and self-doubt, which are already heightened across childhood and adolescence. Inbuilt addictive algorithms^26^ within social media platforms have been linked to social isolation, reduced physical activity and disrupted sleep. Disturbed sleep, in turn, is associated with poorer mental health. At present, there is strong evidence of associations between poor mental health and social media use, but reverse causation and (unmeasured) confounding are also plausible.

### Socioeconomic inequality and marginalized groups

Children from disadvantaged backgrounds continue to face a higher risk of mental health disorders due to the stressors associated with poverty, financial insecurity, poor living conditions, and lack of access to quality education.^27^ During the COVID-19 pandemic, children and young people from the most disadvantaged backgrounds were the most severely affected.^28^ Social networks can suffer from the closure of community resources, e.g. youth clubs and art/sports organisations. Poor mental health literacy and stigma^29^ around mental health continues to deter many from seeking help, although treatment engagement seems to be improving in general.^30^

One of the most alarming trends is the elevation in self-harm and suicide rates among adolescents in marginalised groups, such as LGBTQ+ youth, migrants,^31^ and young people with disabilities.^32^ Socioeconomic inequality and the inclusion of marginalised groups remain one of the principal challenges in child mental health across the WHO Europe region.

### Access to high-quality services

The increased prevalence of mental health disorders is accompanied by a rapid increase in specialist referrals, especially in the aftermath of the COVID-19 pandemic.^20^ Clinical services across Europe (and globally) are not designed to cope with such an enormous disease burden. Such services are unevenly distributed.^33^ Furthermore, the increasing strain on services has led to longer waiting times, more burnout among practitioners, and challenges with clinical recruitment. Funding for child and adolescent mental health remains meagre across the region^34^ despite health-economic analyses indicating that many interventions for children and young people are highly cost-effective.^35^ According to the 2017 survey of healthcare providers in 27 European countries, 16 rated facilities providing community outpatient mental health care to children and adolescents as absent or insufficient.^36^

Even when services are accessed, the quality of care received is variable due to differing levels of user participation in the design and delivery of the service, human rights challenges, long waiting times to access care, and poor provider support and training, and a weak culture of quality improvement.^37^ Only 19 out of 28 countries surveyed in 2017 had a standardized assessment of mental health services,^36^ and only respondents from six countries said that there was regular monitoring of treatment outcomes.

### Childhood adversity

Childhood adversity, such as maltreatment (i.e., physical, sexual, and emotional abuse, and physical and emotional neglect), is a prevalent^38^ and an established trans-diagnostic risk factor for psychopathology^39^^,^^40^ with clear meta-analytic evidence of causal effects from quasi-experimental studies.^41^ Childhood maltreatment increases not only the risk of the onset of new disorders, but is also associated with a worse course of illness and response to treatment^42^^,^^43^ Effects on the incidence and prevalence of psychopathology can be mitigated by evidence-based interventions. The impact of maltreatment on psychopathology appears to be explained by an individual's appraisal and memories of such events,^44^, ^45^, ^46^ and trauma-focused cognitive behavioural therapy is effective for treating post-traumatic stress disorder and comorbid symptoms following sexual abuse,^47^ maltreatment^48^ and multiple trauma exposure.^49^ However, most young people who develop trauma-related psychopathology do not access any mental health care.^50^ In 2017, out of 27 countries, ten were reported to provide access to refugees, seven to orphans or victims of natural or man-made disasters, and four to minority groups. Nine of 27 countries had no special services designed to meet the specific needs of these subgroups, and only seven indicated having highly specialised services for fostered children, children who have offended and been charged with crimes, disabled children, children with autism, or children who misuse substances.^36^

### Military conflicts

Wars, such as the war in Ukraine, have had a major impact on the mental health of children and young people in the WHO Europe region.^51^ As a result of these conflicts and others around the world, millions of children and adolescents in the region have been displaced. Moreover, different countries in the WHO Europe region absorbed varying numbers of refugees. Some countries, such as Bulgaria, with relatively poorly developed mental health services, received the greatest share of asylum-seeking applications from unaccompanied minors between 2014 and 2024.^52^ This, in turn, increases treatment gap even further. There is a high prevalence of symptoms of PTSD and other psychiatric disorders, such as depression and addictions, often co-occurring in these young people affected by military conflict.^53^ There is a limited evidence base for treating large numbers of war-affected children and adolescents, especially those who remain inside the conflict zones.^54^

### Limited pharmacological approaches

Psychopharmacological treatment is an important component of the multimodal intervention approach to mental health conditions in children and adolescents. Paediatric Investigation Plans (PIPs) are mandatory for new drugs or indications. However, PIPs have been overall unsuccessful. Psychiatry remains underrepresented in paediatric medicine, comprising only 2.4% of all paediatric therapeutic areas, according to the European Commission's 2017 Ten-Year Report.^55^ The issue is not due to a lack of efficacy or safety issues, as current evidence shows a moderate to high effect size from clinical trials and overall good tolerability. Rather, the issue is due to small market share and pharmaceutical, ethical challenges, and systemic and regulatory barriers in psychopharmacological treatment.

Many psychopharmacological studies in children are either unnecessary or poorly designed. A review found that 22% of paediatric studies could have used better extrapolation methods from adult data, and 12% were entirely redundant.^56^ Tools like physiologically based pharmacokinetics (PBPK) and simulations are underutilised despite their promise in improving study quality. Clinical trials tend to include selected populations of children and young people, which makes translation of the findings difficult as the real-life patients often present with complex comorbidities and high risk.

Many medications are still used off-label (i.e., outside the limits of the marketing authorisation or product license), or their availability is lacking in child and adolescent psychiatric practice. A survey^57^ among the members of the European College of Neuropsychopharmacology- Child and Adolescent Network, the disorders/conditions for which there is a need for additional pharmacological development identified are autism spectrum disorder (core symptoms), emotional dysregulation/irritability, anorexia nervosa and depression as the top four.

Onechallenge in identifying new, effective medications is the placebo effect. For example, the placebo response is generally higher in depression than in anxiety,^58^ and higher in youth depression than in adult depression. Moreover, the placebo response is higher in multi-site studies, which is the norm in youth depression studies, and seems more common in pharmacologically funded than in government-funded trials (a reversal of what one might expect). The placebo response means that treatments with a 60% response rate, such as SRIs, only show marginal efficacy due to poor separation from the placebo condition, although there are exceptions to this rule, such as in Obsessive Compulsive Disorder.^52^

## Possible solutions

Preventative strategies in child and adolescent mental health hold the potential to significantly reduce the burden associated with poor mental health by addressing risk factors, enhancing protective factors, and intervening early to mitigate the progression of disorders^1^^,^^59^^,^^60^ and they could provide a starting point for addressing many current problems Europe's healthcare systems provide an opportunity to develop, implement, and evaluate innovative prevention programs. Primary preventative strategies traditionally distinguish between three levels: *universal* prevention targets entire populations regardless of risk levels, promoting mental well-being and resilience, such as whole-school approaches to prevent suicide attempts^58^; *selective* prevention focuses on groups with an elevated risk of developing mental health problems, such as children in low-income families, neurodivergent children, or those exposed to adverse childhood experiences^61^; and *indicated* prevention is aimed at individuals exhibiting early signs of poor mental health but not meeting diagnostic thresholds.^62^ Preventative strategies should be seen as complementary to other possible solutions. Most problems identified do not have a single solution, and some require a combination of most of the possible solutions identified.

### Use of innovative approaches of delivery to improve access to high-quality community mental health care

There is a pressing need to take an innovative approach to expand access to mental health services for children and adolescents, particularly in underserved regions and populations. Governments in the WHO Europe region should invest in improving the quality of community-based mental health services. High-quality care has been co-defined through WHO Quality Standards for Child and Youth Mental Health Services and refers to quality standards across eight themes, including care that is co-produced with young people, promotes human rights and a focus on quality improvement.^37^ Quality improvement theories have not been widely integrated with mental health yet provide systematic strategies to strengthen care quality.^63^ Standards can be used to define a realistic vision of high-quality service delivery, indicators can assess progress,^64^ and methods can be used to develop innovative approaches.

A good example of such an intervention is parenting support interventions, which have a strong evidence base but may not be accessible to young people who need them the most.^22^ There are also excellent examples from low-resource settings in the European region and beyond in which non-specialists and community members are trained to deliver high-quality assessments and treatments at scale,^65^ such as WHO Early Adolescent Skills for Emotions (EASE).^66^ Furthermore, the implementation of low-intensity interventions, such as Teaching Recovery Techniques,^67^ can prevent mental illness at a low cost. Digital means provide an opportunity to complement traditional services,^68^ offering therapy and counselling online, which can be especially beneficial in rural or underserved areas and areas affected by military conflicts,^69^ although direct evidence of efficacy in these settings is lacking.^70^ Social media has the potential to improve peer-to-peer support, and to be a vehicle to deliver mental health interventions through the use of influencers and improve public mental health.^71^ The introduction of comprehensive machine learning algorithms in primary care can assist in risk prediction for the development of a range of mental health conditions through analysing demographic and healthcare data.^72^

Early childhood programs with investments in perinatal mental health care, parenting support, and early education can significantly contribute to preventing mental health disorders. For instance, the Nurse-Family Partnership program, implemented in parts of Europe, provides targeted support to at-risk families, demonstrating long-term benefits for both parents and children.^73^^,^^74^ Comprehensive policies for the regulation of alternative delivery methods are essential, in particular in relation to the ethical implications of the use of machine learning and non-specialists in healthcare delivery.

### Moving away from institutional care to community care

Many countries in the region have started to move away from institutional psychiatric care to community-based services.^75^ This is particularly the case for the countries receiving European structural funding, in particular those located in Eastern Europe.^76^ At the same time, there are countries like Belgium, where the number of children in institutional care seems to be increasing^76^ Growing evidence indicates that long-term inpatient mental health care is associated with negative social and psychiatric outcomes.^77^ Despite this knowledge, it is estimated that more than one million children in the region live in long-term care institutions, which often include children with significant social problems, intellectual disabilities and young people with youth justice involvement.^78^ Governments should prioritise the development of high-quality community care, including intensive community care services, as alternatives to in-patient admissions.^77^^,^^79^ It is well known that when substitute care (after child maltreatment or parental death or incapacity) is required, young children's development is served better in family-based care than in institutional care, such as orphanages^80^ and is more cost-effective.^80^

### Integration of mental health into school curricula

Schools play a crucial role in early intervention and prevention of mental health problems. Implementing universal school-based socio-emotional learning programs^35^ can help reduce stigma, foster emotional resilience, and provide students with coping strategies. Evidence-based programs like MindMatters, Social and Emotional Aspects of Learning (SEAL), and SPARK Resilience have been widely adopted across European countries (EU, 2024).

Many experts recommend including suicide prevention programs, peer support initiatives, and training for teachers to recognise signs of distress among students.^58^ Yet, there seems to be a high degree of variability in terms of priority given to mental health provision in schools, with 72.8% of schools in Poland reporting mental health provision to be a priority, and only 17.8% in France.^81^ Interestingly, even in countries where national policy recognises the importance of mental health support in schools, such as the United Kingdom and the Netherlands, only 59.8% and 52.9% of schools rate mental health as a priority. Only 15.7% of schools in France had a dedicated mental health policy, in comparison to 78.3% in the Netherlands. Based on the findings of the classic Adverse Childhood Experiences (ACE) Study,^82^ population screening for ACEs has been implemented at scale in different countries. However, several concerns have been raised about this rapid implementation of ACE screening, and improved methods are required to improve risk detection and deliver targeted interventions.^83^ Implementation of screening in general should be planned carefully, and any potential harm and increased stigmatisation should be taken into account. Individual risk modelling approaches should be employed to improve the accuracy of screening tools in predicting mental health disorders and to guide the selection of individualised interventions.^81^

### Addressing socioeconomic determinants of mental health

Tackling mental health issues requires addressing the broader social determinants that contribute to psychological stress. Policies aimed at reducing child poverty,^84^, ^85^, ^86^ improving housing conditions,^87^ and ensuring equitable access to healthcare and education^86^ can reduce the risk of mental illness. Addressing social determinants of mental health is not the responsibility of the health sector alone; intersectoral collaboration in the development of policy and programs is required–across education, housing, employment and training, and social welfare. Healthcare professionals and practitioners should be trained to recognise the role of social determinants in the course of mental health difficulties and consider how best to address these. Conversely, practitioners should not be blinded to psychiatric conditions in young people who have experienced adversity, since this can lead to missed or late diagnoses.^88^ Furthermore, special attention must be given to marginalised groups such as refugees, asylum seekers and displaced persons, as well as those from LGBTQ+ and ethnic minority groups,^89^ ensuring they have tailored support systems that consider their specific needs.

### Co-designing and Co-regulating digital spaces

Involving young people in co-design, co-production and co-development of policies and regulations for digital spaces is imperative.^90^ Given the role of social media in shaping adolescent mental health, governments and digital platforms must work together to create safer online environments. WHO Europe has developed guidance on how governments can address the impacts of social media and digital technologies on young people's mental health and wellbeing through eight priority actions.^83^ These include holding industry to account for harmful platform features, stronger regulation aimed at protecting health and well-being, and investment in offline alternatives to screen-based parenting, play, and entertainment. Initiatives to raise awareness about the risks of cyberbullying and the negative impact of excessive screen time are essential.

### Strengthening family and community support systems

Given the importance of social connections in increasing well-being and the negative impacts of loneliness across a range of adolescent mental health conditions,^91^ it is crucial to foster access to social support and resources in children and young people. Beyond schools and health services, extended families and communities are critical in fostering a supportive environment for children and adolescents. Inspired by longstanding initiatives such as the Healthy Cities network, countries across the region are increasingly exploring ways to enhance the capacity of cities and neighbourhoods to promote and protect the mental health of young people through interventions delivered locally, informed by local needs and drawing on local resources.^92^ Research on neighbourhoods has shown that structural elements, such as household income, and process elements, such as social cohesion, are important determinants of child mental health.^93^ This is especially true of those affected by wars, where supportive relationships and social participation can be crucial resilience factors.^94^ Governments should support community-based mental health initiatives that offer parenting support programs, programs that support food security and food quality and peer mentorship. An example of this is social prescribing,^95^ a referral mechanism in which a child/young person is linked to local organisations that coordinate and facilitate activities known to benefit well-being, such as arts, sports and nature. In social prescribing, children and young people have agency over which activities are chosen, and so this approach may be experienced as empowering, more engaging and less stigmatising than other treatments. One size will not fit all, and initiatives to improve social connectedness must be tailored to different geospatial and social contexts.

### Further development of pharmacological treatments

Overall, international pharmaco-epidemiological data point to increased use of psychotropic medications for children/adolescents in the past two decades.^96^ An increasing body of evidence has been generated from randomised controlled trials (RCTs) and observational studies assessing the efficacy/effectiveness and tolerability/safety of medications for specific disorders in child/adolescent mental health. Overall, investigated medications show moderate to high effect sizes from short term RCTs (Table 1).

**Table 1:** Psychotropic medications approved by the European Medicine Agency (EMA), grouped by indication in alphabetical order. Medications are presented by condition (in alphabetical order).

Compound	Indication (note: national approvals are not included)	Age (years)	Effect size^a^ (95% CI)
Guanfacine, extended release	ADHD	6–17	−0.67 (−0.85 to −0.50)
Methylphenidate	ADHD^a^	>6 years	−0.78 (−0.93 to −0.62)
Atomoxetine	ADHD	>6 years	−0.56 (−0.47 to −0.65)
Aripiprazole	manic episodes	≥13 years	−1.08 (−1.32 to −0.85)
Risperidone	Conduct disorder	5–18	−0.48 (−0.71 to −0.24)
Fluoxetine	major depressive episode unresponsive to psychotherapy		−0.51 (−0.84 to −0.18)
Melatonin extended release	*Insomnia (in ASD or Smith Magenis syndrome)*	2–18	Sleep latency: −0.52 (95% CI not reported)
Sodium oxybate	Narcolepsy	≥7	Not available
Sertraline	*Obsessive Compulsive Disorder*	6–17	−0.24 (−0.46 to −0.03)
Aripiprazole	schizophrenia	≥15 years	−0.43 (−0.63 to −0.24)
Lurasidone	schizophrenia	≥13 years	−0.48 (−0.71 to −0.25)
Paliperidone	schizophrenia	≥15 years	−0.42 (−0.66 to −0.18)

a Effect sizes are based on the umbrella review by Correll et al.^97^; when effect sizes derived from scored by multiple raters are available, clinician raters are reported; the effect size for melatonin is based on Gringras et al.^98^; effect size for sodium oxybate are not reported in the publication of the RCT by Plazzi et al.^99^

The highest effect sizes in relation to efficacy have been found^97^ for stimulants for core symptoms of ADHD; aripiprazole and risperidone for irritability in autism spectrum disorder (ASD); risperidone for aggressiveness in disruptive behaviour disorders (although this remains an unlicensed indication); risperidone, olanzapine, paliperidone for symptoms of schizophrenia; fluoxetine for depression (the effect size remains uncertain); aripiprazole for manic symptoms in bipolar disorder; fluoxetine/other selective serotonin reuptake inhibitors (SSRIs) for anxiety and obsessive-compulsive disorder (OCD); and imipramine for enuresis. Psychopharmacological medications approved—as of November 2024- by the European Medicine Agency (EMA) in Europe for mental health conditions in children/adolescents are reported in Table 1. This reflects only a small portion of medications approved for mental health conditions in children/adolescents in Europe because companies can apply for registration nationally rather than to the EMA. Furthermore, many medications were licensed before the establishment of the EMA in 1995.

The European Medicines Agency developed an action plan aimed at enhancing the implementation of the regulation and providing structured guidance on the use of extrapolation from adult data to paediatric populations. This plan addresses potential obstacles to paediatric development by focusing on: 1) identifying unmet medical needs in children; 2) strengthening collaboration among decision-makers; 3) ensuring the timely completion of Paediatric Investigation Plans (PIPs); 4) improving the processing of PIP applications; and 5) increasing transparency regarding paediatric medicines.

The main problem in psychopharmacology is that high safety and efficacy standards have increased, requiring costly new studies to meet them. However, for many psychotropic substances, patents have expired, leaving little financial incentive for pharmaceutical companies to invest in pediatric research or apply for new licenses. Without potential profit, they avoid costly trials, particularly for generic medications. Public funding and regulatory incentives are needed to fill these gaps, especially in large-scale safety studies. Overall, key opportunities in child and adolescent psychopharmacology^57^ include learning from failed trials, reducing the placebo effect issue, assessing outcomes beyond core symptoms, considering developmental stage, comparing pharmacological and non-pharmacological treatments, using innovative designs beyond standard RCTs, moving towards precision medicine/stratification approaches, additional investigation and implementation of digital technologies, focusing on conditions that are non-responsive to initial treatment.

### Robust research and implementation strategies

Despite their potential, there remain several opportunities in developing and implementing preventative and treatment strategies for child and adolescent mental health. Some preventative interventions with a strong a priori rationale have proven ineffective or detrimental for some outcomes.^100^ De-implementation might be required for these interventions. There is increasing evidence that psychological intervention studies rarely measure harm, despite evidence that they may cause harm as well as positive effects.^101^ A recent RCT of a whole school-based mindfulness intervention programme, for example, demonstrated no overall effects and suggested possible harm on some outcomes.^100^ In future research studies, a framework for assessing harms and benefits is essential, with some preliminary results in relation to a comprehensive assessment of positive and negative outcomes of health information campaigns.^102^ Any interventions need to be rigorously tested for efficacy and effectiveness and implementation potential, such as acceptability, feasibility and contextual constraints before roll-out, to prevent wasting resources and to balance harms and benefits. Moreover, it is vital to understand who are the people targeted by each intervention. Most notably, anti-depressant trials often exclude people with significant suicidality. Yet, these are the patients in most need and arguably the ones clinicians are most likely to treat with SSRIs. The effect sizes for preventative interventions are often small, yet can have vast public implications and might be associated with helpful shifts in population mental health.^103^ However, the cost-effectiveness of these strategies must be carefully evaluated.

The establishment of unified protocols for treatment and prevention requires a careful comparison between the different evidence-based approaches. Psychological therapy is often the first-line treatment for depression in children and adolescents. Yet, studies showing its effectiveness are methodologically very different to those done for medication, they are non-blinded and often have poorly matched control groups,^104^ making comparison between the outcomes from these approaches very difficult.

Equally, interventions which do demonstrate significant improvements need to be implemented rapidly, consistently and widely.^1^ For example, the SEYLE, (Saving and Empowering Young Lives in Europe) indicated that the Youth Aware of Mental Health (YAM) program can substantially reduce the risk of attempted suicide and is cost-effective. Yet, the implementation of this program has been patchy at best.^105^ It is important that interventions and care pathways should be co-designed and co-created with relevant stakeholders, e.g. policymakers, teachers, parents and most importantly, children and young people. This will ensure that implementation takes into account the nuances of contexts and individual needs and preferences. The involvement of stakeholders will be critical to map and address key barriers to engagement with child and adolescent mental health services, including, for example, stigma, limited mental health literacy, negative beliefs towards mental health services, costs to access services, differential service access for marginalised groups,^106^ limited mental health training in primary care, limited availability of specialist mental health professionals, and high demand for clinical care.^107^, ^108^, ^109^

## Discussion

Prevention of mental ill health remains a priority; however, there are regional differences in the available systems designed to deliver it. Some areas rely on the traditional medical system, while others have a much more developed public health system. If the latter is the case, then creating a preventive or health-promoting environment from birth to adulthood can be assisted by expertise from child and adolescent psychology, psychiatry and allied professions, maintaining a holistic, rather than symptom-based focus. The regional analysis of 38 national mental health policies and plans show considerable variation.^110^ While a top-down national model is predominant in Eastern Europe, a bottom-up locally driven model is more frequent in Northern Europe. Only five countries had established multi-sectoral bodies for MHPP implementation. Communication processes between sectors were generally underdeveloped. In many countries in the East of the WHO Europe region, Ministries of Health alone were tasked with the implementation of MHPPs. In many Western and Northern parts, other governmental stakeholders, as well as non-governmental bodies and service user organisations, were incorporated as implementation partners.

This article represents an overview of the key elements of clinical and research challenges, opportunities and recommendations relevant to the mental health of children and young people in the WHO Europe region. Many of these apply to all countries and regions, yet the high diversity within Europe regarding health care and welfare systems, school systems and income inequalities needs to be recognised. A lot of the recommended programmes still need to be implemented. Barriers to implementation vary by region, from low resource availability in some regions to rigid configuration of services in others.

The paper represents a policy-oriented contribution, although we attempted to rely on previously published systematic reviews and meta-analyses to the best of our knowledge.

## Conclusion

The mental health challenges among children and adolescents in the WHO Europe region are multifaceted and driven by social, economic, environmental and technological factors. Addressing this issue requires a holistic approach that includes evidence-based preventive measures in schools, equitable access to mental health services, ending wars and concerted efforts to tackle the root causes of socioeconomic inequality. In recognition of the great diversity of culture and contexts across the WHO Europe region, the implementation of these strategies should be tailored for each context, co-developed with local experts and service users. Since the vast majority—if not all-countries are struggling to respond to the mental health needs of their children and adolescents, there is also opportunity for cross-country collaborations to jointly solve problems and share innovations. By promoting a culture of understanding and support for mental health, the region can take significant steps toward safeguarding the well-being of its younger generations.

## Contributors

All authors contributed equally.

## Declaration of interests

Authors declare no competing interests.
